# An audit of the management of childhood-onset growth hormone deficiency during young adulthood in Scotland

**DOI:** 10.1186/s13633-016-0024-8

**Published:** 2016-03-16

**Authors:** M. Ahmid, V. Fisher, A. J. Graveling, S. McGeoch, E. McNeil, J. Roach, J. S. Bevan, L. Bath, M. Donaldson, G. Leese, A. Mason, C. G. Perry, N. N. Zammitt, S. F. Ahmed, M. G. Shaikh

**Affiliations:** Developmental Endocrinology Research Group, Royal Hospital for Children, School of Medicine, University of Glasgow, 1345 Govan Road, Glasgow, G51 4TF UK; JJR Macleod Centre for Diabetes, Endocrinology & Metabolism, Aberdeen Royal Infirmary, Aberdeen, UK; Department of Endocrinology, Royal Hospital for Sick Children, Edinburgh, UK; Ninewells Hospital and Medical School in Dundee, Dundee, UK; Department of Endocrinology, Queen Elizabeth University Hospitals, Glasgow, UK; Royal Infirmary of Edinburgh, Edinburgh, UK

**Keywords:** Childhood onset growth hormone deficiency, Retesting, Re-evaluation, Transition, Adolescence

## Abstract

**Background:**

Adolescents with childhood onset growth hormone deficiency (CO-GHD) require re-evaluation of their growth hormone (GH) axis on attainment of final height to determine eligibility for adult GH therapy (rhGH).

**Aim:**

Retrospective multicentre review of management of young adults with CO-GHD in four paediatric centres in Scotland during transition.

**Patients:**

Medical records of 130 eligible CO-GHD adolescents (78 males), who attained final height between 2005 and 2013 were reviewed. Median (range) age at initial diagnosis of CO-GHD was 10.7 years (0.1–16.4) with a stimulated GH peak of 2.3 μg/l (0.1–6.5). Median age at initiation of rhGH was 10.8 years (0.4–17.0).

**Results:**

Of the 130 CO-GHD adolescents, 74/130(57 %) had GH axis re-evaluation by stimulation tests /IGF-1 measurements. Of those, 61/74 (82 %) remained GHD with 51/74 (69 %) restarting adult rhGH. Predictors of persistent GHD included an organic hypothalamic-pituitary disorder and multiple pituitary hormone deficiencies (MPHD). Of the remaining 56/130 (43 %) patients who were not re-tested, 34/56 (61 %) were transferred to adult services on rhGH without biochemical retesting and 32/34 of these had MPHD. The proportion of adults who were offered rhGH without biochemical re-testing in the four centres ranged between 10 and 50 % of their total cohort.

**Conclusions:**

A substantial proportion of adults with CO-GHD remain GHD, particularly those with MPHD and most opt for treatment with rhGH. Despite clinical guidelines, there is significant variation in the management of CO-GHD in young adulthood across Scotland.

## Background

The transition of care from childhood to adulthood for many chronic disorders requires a careful coordinated approach and this is particularly important in growth hormone deficiency (GHD). Traditionally, children with childhood onset GHD (CO-GHD) discontinue recombinant human GH therapy (rhGH) after attaining final height. However, adults with CO-GHD may have increased fat mass, decreased muscle mass and low bone mineral density, as well as reduced cardiac performance, altered lipid status, reduced physical performance, impaired cognitive function and reduced well-being [1, 2]. Reports suggest that these adults may benefit from rhGH [3, 4]. A number of studies have shown that a high proportion of CO-GHD patients remain GH deficient as adults especially those with multiple pituitary hormone deficiencies (MPHD) and/or structural abnormalities, whereas the majority of those with idiopathic or isolated GHD no longer have GHD in adulthood [5–7]. Therefore, after childhood treatment it is necessary to review GH status in order to assess appropriateness of adult rhGH replacement [8]. However, the extent of benefit from this therapy may be variable and the decision to reinstitute rhGH needs to be undertaken carefully.

In this context, clinical practice guidelines have been issued on the subject of transition of care of young adults with CO-GHD [9–12]. However, the practicalities of these guidelines as well as the extent to which these guidelines have been implemented in clinical practice are unclear. The purpose of this multicentre study was to understand the variation that may exist in the management of young adults with CO-GHD after attainment of final height.

## Methods

We reviewed databases from the four specialist endocrine centres in Scotland and identified young adults who had been diagnosed as having CO-GHD and who had been treated with GH during childhood and had subsequently reached final height between 2005 and 2013. Study entry criteria were: CO-GHD (low GH peak response on stimulation test <6.6 μg/l), GH treatment during childhood, attainment of final height between 2005 and 2013 (height velocity <1 cm/year as defined in all centres), and evaluation of GH- axis by stimulation tests and/or IGF-1 levels after withdrawal of GH for at least one month. Exclusion criteria included: untreated CO-GHD, GH-treated patients with CO-GHD who have not yet attained final height. Baseline demographic data included: aetiology of CO-GHD, age at diagnosis of CO-GHD, duration of GH treatment, presence of multiple pituitary hormone deficiencies (MPHD), re-evaluation of GH axis, and whether adult GH treatment was recommenced or not. The persistent GHD after retesting for four centers was defined as cutoff <5 μg/L GH peak response for dynamic stimulation testes and/or low serum IGF-1 levels (<2 SD for age and sex) [9]. IGF-1 level measurement for centres A, B, and D were done using IDS iSys and centre C measured IGF-1 levels by immunoassay on the Siemens Immulite. All IGF-1 levels were corrected for age and sex accordingly.

### Statistical analysis

Data were analyzed using Minitab software (Version 16) with a significance level of <0.05 and are described as median, ranges and percentage. Additionally, the Mann–Whitney U–test was used for calculation of significance of differences between median values. Association with clinical factors was assessed by Spearman’s rank coefficient and a positive predictive value (PPV) was calculated for the identified predictors of persistent GHD.

## Results

### General characteristics

A total of 142 patients were screened, 130 of whom met inclusion criteria. The 130 patients (78 male) comprised of: 70 from centre A, 32 from B, 18 from C and 10 from D. Table 1 displays the aetiology of CO-GHD. Median age at diagnosis of CO-GHD was 10.7 years (0.1–16.4) with an initial stimulated GH peak of 2.3 μg/l (0.1–6.5), and basal IGF-1 was 74 μg/l (4.0–410.0). Median age at initiation of rhGH was 10.8 years (0.4–17.0). GH peak at diagnosis was lower in those with MPHD compared to IGHD (1.9 μg/l (<0.1–6.4) vs 3.0 μg/l (0.3–6.5) respectively: *p* < 0.01).

**Table 1:** The categories of patients with CO-GHD according to aetiology and centres distribution is shown as (A, B, C, D)

	Total number of cases 130	IGHD	MPHD*
		48/130 (37 %)	82/130 (63 %)
Congenital *n* (%) (A,B,C,D)	38/130 (29 %)	12 (8,1,2,1)	26 (14,3,4,5)
-Pituitary axial structural abnormalities (A,B,C,D)	24	9 (6,1,1,1)	15 (6,3,1,5)
-Midline axial structure defects (SOD) (A,B,C,D)	14	3 (2,0,1,0)	11 (8,0,3,0)
Oncology/cranial irradiation *n* (%) (A,B,C,D)	51/130 (40 %)	8 (5,3,0,0)	43 (18,19,4,2)
- Craniopharyngioma (A,B,C,D)	15	–	15 (6,7,1,1)
- Hematologic malignancies (A,B,C,D)	12	4 (4,0,0,0)	8 (6,0,1,1)
- Medulloblastoma (A,B,C,D)	6	1 (0,1,0,0)	5 (1,4,0,0)
- Other CNS tumors (A,B,C,D)	18	3 (1,2,0,0)	15 (5,8,2,0)
Idiopathic^a^ *n* (%) (A,B,C,D)	15/130 (11 %)	13 (7,1,5,0)	2 (1,1,0,0)
Others^b^ *n* (%) (A,B,C,D)	26/130 (20 %)	15 (12,1,1,1)	11 (5,3,2,1)
-Crohn's disease (A,B,C,D)	4	4 (3,0,0,1)	–
-Coeliac disease (A,B,C,D)	2	–	2 (0,1,1,0)
-Haematological diseases^c^ (A,B,C,D)	2	1 (1,0,0,0)	1 (1,0,0,0)
-Other diseases^d^ (A,B,C,D)	11	8 (6,1,1,0)	3 (0,2,1,0)
-Syndromes^e^(A,B,C,D)	6	2 (2,0,0,0)	4 (3,0,0,1)
-Acquired brain injury (A,B,C,D)	1	–	1 (1,0,0,0)

Data are presented as the numbers of patients and percentages are given in *parentheses*

*33/82 patients with one additional pituitary hormone deficiency, 17/82 with two additional deficiencies, 19/82 with three and 13/82 with four additional deficiencies ‘panhypopituitarism’

IGHD, isolated growth hormone deficiency; MPHD, multiple-pituitary hormone deficiencies; SOD, Septo-optic dysplasia

^a^Normal pituitary MRI, GHD is not associated with other conditions

^b^Normal pituitary MRI (or no MRI report), but GHD is associated with other conditions

^c^(Thalassemia, X-linked Sideroblastic Anaemia)

^d^(Microephaly with learning disability, history of intrauterine growth retardation, gastrochisis with history of small for gestational age, Asthma, joint hypermobility syndrome, pesudohypoparathyrodism)

^e^(Charge syndrome, Noonan syndrome, Kallman Syndrome, trisomy 22, Klinefelter's syndrome, Turner's syndrome with GHD)

### Re-evaluation of GH axis

**Fig. 1:**
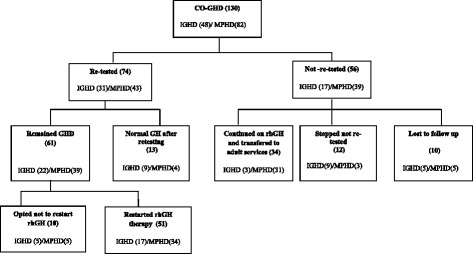
Study Cohort Flow Chart. (n); number of patients, CO-GHD; childhood onset growth hormone deficiency, IGHD; isolated growth hormone deficiency, MPHD; multiple-pituitary hormone deficiencies, GH; growth hormone therapy

A total of 74/130 (57 %) patients with CO-GHD (IGHD = 31 (42 %): MPHD = 43(58 %)) were biochemically retested at a median age of 18.2 years (14.5–21.3) (with one outlier patient who was retested at the age of 27.5 years), rhGH treatment was discontinued at the median age of 16.4 years (10.8–21.0). Biochemical retesting was performed after a median period of 0.5 years (0.1–5.6) off rhGH (21/74 (28 %) were retested over period of (0.1–0.3 years) and 34/74(46 %) over a period of (0.4–5.7 years), with incomplete data on timing of re-testing in 20/74 (27 %). Median duration of childhood treatment was 5.3 years (0.4–16.8). At retesting, the median GH peak was 1.6 μg/l (0.1–23.7) and IGF-1 was 88.0 μg/l (15.0–631.0). Of those retested, 61/74 (82 %) (32 males) remained GHD and were eligible for adult rhGH, with 51/61 (84 %) re-starting adult rhGH and 10/61 (16 %) declining therapy although it is possible that they may have restarted at a later stage. The remaining 13/74 (18 %)(10 males) who were no longer GH deficient consisted of eight with idiopathic IGHD, two brothers with central hypothyroidism and normal pituitary MRI, one with an ectopic pituitary, one with hypogonadism and Coeliac disease and one with a history of cranial irradiation. Of the 56 of 130 (43 %) cases of CO-GHD who were not retested 34 (61 %) were transferred from paediatric to adult services without biochemical retesting during transition, 12 (21 %) stopped treatment without biochemical re-evaluation and 10 (18 %) were lost to follow up whilst on treatment (Fig. 1).

Dynamic function stimulation tests were performed in 40/74 (54 %) patients who were retested, with 35/40 (88 %) of subjects having a low GH peak response <5 μg/l, with 27/35 of them having severe GHD with a GH peak response <3 μg/l. Of the remaining 34/74 (46 %) patients who were retested, IGF-1 levels alone were available and low enough to confirm GHD (≤ -2 SD for age and gender) in 19/34 (56 %) of which 15 had MPHD and 4 had IGHD (organic causes and abnormal pituitary MRI). Two patients (MPHD) had IGF-1 levels within normal range on initial retesting (>2 SD for age and gender), but were confirmed to have GHD following GH stimulation tests.

### Reconfirmation of GHD and initiation of adult GH replacement therapy

Of the 130 with CO-GHD, 34 (26 %) patients continued adult rhGH without temporary cessation of therapy or formal retesting. Of these 34, nine had structural abnormalities on MRI, 22 were related to late effects of cancer therapy and three had unexplained GHD. Of those 34, 31 (91 %) had MPHD (17/32 of them had three or more additional pituitary hormone deficiencies (PHDs)); and 3/34 (9 %) had IGHD (two with pituitary structural abnormalities on MRI and one with tumour related GHD). These patients were advised to continue rhGH until their mid-20s.

For patients who were re-tested, GH cut offs for considering rhGH varied between centres. Not all patients found to have persistent GHD restarted adult GH therapy despite low peak GH levels at re-testing. There were four patients who were found not to have severe GHD with GH peaks 4-5 μg/l (three patients from centre B, one from centre A) and were not offered rhGH as they did not meet adult criteria for replacement. However, among those who were offered rhGH after retesting, one patient with IGHD (centre C) had a GH peak >5 μg/l (5.5 μg/l).

### Variation in the management between centres

There were substantial variations in the management of CO-GHD between Scottish centres. Re-testing with stimulation testes and/or IGF-1 levels was found to be the highest in centre A (68 %), while centre C had the lowest percentage of retested patients (28 %), although this did include all IGHD patients from centre C. Centre B did not retest those with a high likelihood of permanent GHD (especially those with three or more additional PHDs) and had the highest percentage of adults on rhGH without biochemical re-evaluation (Table 2). A total of 32/130 patients in the cohort had three or more additional PHDs (Table 3). Of these 32, 14 (44 %) were retested using their IGF-1 levels alone and all were confirmed to have adult GHD, 17 (53 %) continued on rhGH without biochemical retesting and one was lost to follow up whilst on treatment.

**Table 2:** Management of patients with CO-GHD according to each Scottish centre

	All centres	A	B	C	D
Total number of patients (*n*)	130	70	32	18	10
Total number of patients re-tested *n* (%)	74/130 (57)	48/70 (69)	16/32 (50)	5/18 (28)	5/10 (50)
Persistent GHD *n* (%)	61/74 (82)	43/48 (90)	12/16 (75)	1/5 (20)	5/5 (100)
Those with persistent GHD who restarted rhGH *n* (%)	51/61 (83)	35/43 (81)	11/12 (92)	1/1 (100)	4/5 (80)
Total number of patients *not*-retested *n* (%)	56/130 (43)	22/70 (31)	16/32 (50)	13/18 (72)	5/10 (50)
Continued adult rhGH therapy without re-testing *n* (%)	34/56 (61)	7/22 (32)	16/16 (100)	7/13 (54)	4/5 (80)
Lost to follow up whilst on treatment *n* (%)	10/56 (18)	9/22 (41)	0	0	1/5 (20)
Stopped GH, no re-testing required *n* (%)	12/56 (21)	6/22 (27)	0	6/13 (46)	0

Data are presented as the numbers of patients and percentages are given in *parentheses*

**Table 3:** Variation in the management of patients with CO-GHD between the four Scottish centres according to GHD categories

Centres	A		B		C		D	
	IGHD	MPHD	IGHD	MPHD	IGHD	MPHD	IGHD	MPHD
Total CO-GHD *n* = 130 [32]	32	38[16]	6	26[10]	8	10[3]	2	8[3]
Retested *n* = 74 [14]	21	27[13]	4	12[0]	4	1 [0]	2	3[1]
With structural abnormalities^a^	8	13[5]	–	2	1	–	–	–
Tumour related^b^	3	13[8]	2	7	–	1	–	1
Idiopathic/unexplained^c^	10	1	2	3	3	–	2	2[1]
Re-confirmed GHD	16	27[13]	3	9[0]	1	0	2	3[1]
Not retested (*but on adult rhGH*) *n* = 34 [17]	0	7[2]	2	14[10]	1	6 [3]	–	4[2]
With structural abnormalities^a^	–	–	2	1	1	3 [3]	–	1
Tumour related^b^	–	5[2]	–	12[10]	–	3	–	1[1]
Idiopathic/unexplained^c^	–	2	–	1	–	–	–	2[1]

Data are presented as number of patients with CO-GHD and [number of patients who have three and more additional pituitary hormones deficiencies

^a^MRI imaging reported hypothalamic-pituitary axial structural abnormalities

^b^Cranial irradiation

^c^Normal pituitary MRI/Congenital GHD unexplained (no MRI report)/and/or associated with chronic disease

### Predictors of persistent GHD on re-evaluation

Patients with persistent GHD were diagnosed at an earlier age ((8.4 years (0.3–16.0) vs 11.6 years (7.1–15.5)) and reached final height with re-evaluation of their GH axis in earlier adolescence ((17.9 years (14.2–21.2) vs 19.3 years (17.3–21.3)) than those who were no longer GH deficient on retesting. No significant differences in the other parameters between persistent GHD and non-persistent GHD were identified at time of diagnosis or re-evaluation. In this population the peak GH level on retesting was positively related with the GH peak level at childhood (*r* = 0.4, *P* = 0.02). The number of additional PHDs was a predictor of a low peak GH on retesting as all patients with two or more additional PHDs had a lower GH response (<5 μg/l) at reassessment with a PPV (93 %). The presence of hypothalamic–pituitary structural abnormalities has a high PPV (96 %) of persistent GHD, as of the 25 who were retested, 24 were reconfirmed with persistent GHD. Similarly, CO-GHD with a history of cranial irradiation predicted persistent GHD in adulthood (96 %).

## Discussion

Our data confirm that a high proportion (82 %) of the retested patients with CO-GHD continue to have GHD as adults. The majority (80 %) of those who remain GH deficient opted to resume adult GH treatment, however it is unknown whether they complied with therapy and for how long they continued with the treatment. It may also be the case that those adults who were GHD initially declined to restart rhGH during transition, but later reconsidered GH therapy. Factors influencing this decision would be an important area for future studies.

Previous published studies have reported variable estimates of persistent GHD in adulthood ranging from 12.5–90 % [13, 14] but the high incidence of ongoing GHD in adulthood in our cohort may be attributed to the large proportion of patients with organic causes for their GHD. Some of our subjects with MPHD who had no structural abnormalities on MRI continued to have GHD, raising the possibility of an underlying genetic disorder. Similarly, the majority of idiopathic IGHD who were re-evaluated were GH deficient which may also indicate an underlying genetic predisposition. These findings suggest the importance of follow up and regular assessment of pituitary function in those with a low probability of persistent GHD, as they may develop other pituitary hormone deficiencies as previously demonstrated [15, 16]. On the other hand, some patients who would be considered as having a moderate to high probability of persistent GHD (IGHD with structural abnormalities, patients with MPHD or those with a history of cranial irradiation) were no longer GH deficient. These findings demonstrate the limitations in using “at risk” groups to determine who require re-evaluation of their GH axis and those who do not.

Our data confirm that while there are no unequivocal auxological or clinical signs that predict the transiency or the persistence of GHD, a history of organic disease, the presence of two or more additional PHDs [17, 18], presence of hypothalamic-pituitary structural abnormalities and tumour related organic GHD are strong indicators of persistence GHD [19–22].

In terms of timing of retesting, the current guidelines suggest that a period from one to three months off rhGH is sufficient for retesting [9]. Our data show a variable interval between stopping treatment and reassessment, with only 28 % of patients off rhGH for less than 3 months. It is not clear for those who were off rhGH for longer duration before reassessment whether their stopping rhGH was for reasons other than re-testing. However, this prolonged period off rhGH may be associated with detrimental effects on somatic bone and body composition development during transition [23, 24], with recommendations for prompt resumption of rhGH in individuals with clinical evidence of persistent GHD [25]. Furthermore, a longer interval off rhGH may increase the risk of being lost to follow up in these already vulnerable patients and continued follow up around this time is essential [26]. We recommend that in patients who are under the care of paediatric services, the evaluation of GHD in transition should be undertaken by the paediatric clinic, ideally in the context of a joint transition service to improve the follow up and smooth transfer of adolescents with chronic endocrine conditions to the adult services as previously suggested [27].

The principle of offering rhGH during transition for those who have ongoing severe deficiency was variable between centres as the cut-offs chosen are variable, though the majority were in keeping with the guidance suggesting a GH peak <5 μg/l constituting severe GHD in transition [9, 10]. Few patients in our cohort declined restarting adult rhGH, they may be asymptomatic and therefore reluctant to restart rhGH therapy. Approximately one third of our subjects were considered to be very likely to have permanent GHD and therefore continued rhGH uninterrupted, apart from adjustment to an adult GH dose. This is in line with current guidelines which recommend that patients with severe congenital or acquired panhypopituitarism with three or more pituitary hormone deficiencies or identified genetic mutations may not require re-evaluation of their GH status; otherwise all patients with CO-GHD require biochemical re-evaluation and reconfirmation of GHD during transition before reinstituting adult GH replacement therapy [9, 12]. However, of the 34 patients who continued adult rhGH without formal retesting, nine had structural abnormalities on MRI probably were at high risk of ongoing GHD, but three had unexplained GHD and probably should have been retested. Furthermore, some centres still retested those with a high likelihood of permanent GHD, by checking their IGF1 levels, although all were reconfirmed GHD and resumed rhGH. On these grounds, it seems that no clear consensus has been reached as to whether or not to withdraw treatment and retest those at high risk of permanent GHD. It is also unclear whether those who continued rhGH without biochemical re-testing were re-evaluated at a later stage. For those who restarted rhGH, according to the current guidelines, at completion of somatic growth (approximately 25–30 years old) further re-evaluation should be undertaken with the offer of adult GH replacement therapy and monitoring in accordance with National Institute for Health and Care Excellence guidance (NICE) (TA 64 August 2003).

## Conclusion

In conclusion, this study not only provided a snapshot of the differences in management of CO-GHD during transition across Scotland, but it has also enabled us to identify areas of uncertainty despite there being clinical practice recommendations. Our data showed a substantial proportion of patients with CO-GHD remain GH deficient and most opt for rhGH as adults, although not all patients may require re-evaluation of their GH axis. This study also raises concerns about follow up of those who no longer have GHD and patients with GHD who opted not to resume adult rhGH. The optimal management of adolescents with CO-GHD requires continuous follow up during transition and effective communication between paediatric and adult services.

## Abbreviations

CO-GHD: childhood onset growth hormone deficiency
GH: growth hormone
GHD: growth hormone deficiency
IGF-1: insulin-like growth factor 1
IGHD: isolated growth hormone deficiency
MPHD: multiple pituitary hormone deficiencies
MRI: magnetic resonance imaging
PPV: positive predictive value
SDS: standard deviation score
